# Spatially explicit analysis reveals complex human genetic gradients in the Iberian Peninsula

**DOI:** 10.1038/s41598-019-44121-6

**Published:** 2019-05-24

**Authors:** João Pimenta, Alexandra M. Lopes, Angel Carracedo, Miguel Arenas, António Amorim, David Comas

**Affiliations:** 1 Instituto de Investigação e Inovação em Saúde, Universidade do Porto, Porto, Portugal; 2 Institute of Molecular Pathology and Immunology of the University of Porto, Porto, Portugal; 3 Institute of Evolutionary Biology (CSIC-UPF). Departament de Ciències Experimentals i de la Salut, Universitat Pompeu Fabra, Barcelona, Spain; 4 Faculty of Sciences, University of Porto, Porto, Portugal; 5 Instituto de Ciencias Forenses, Universidade de Santiago de Compostela, Santiago de Compostela, Spain; 6 Grupo de Medicina Xenómica, CIBERER, Santiago de Compostela, Spain; 7 Department of Biochemistry, Genetics and Immunology, University of Vigo, Vigo, Spain; 8 Biomedical Research Center (CINBIO), University of Vigo, 36310 Vigo, Spain; 9 Present Address: Centro de Investigação em Biodiversidade e Recursos Genéticos, InBIO Laboratório Associado, Universidade do Porto, Vairão, Portugal

**Keywords:** Genetic variation, Biological anthropology

## Abstract

The Iberian Peninsula is a well-delimited geographic region with a rich and complex human history. However, the causes of its genetic structure and past migratory dynamics are not yet fully understood. In order to shed light on them, here we evaluated the gene flow and genetic structure throughout the Iberian Peninsula with spatially explicit modelling applied to a georeferenced genetic dataset composed of genome-wide SNPs from 746 individuals belonging to 17 different regions of the Peninsula. We found contrasting patterns of genetic structure throughout Iberia. In particular, we identified strong patterns of genetic differentiation caused by relevant barriers to gene flow in northern regions and, on the other hand, a large genetic similarity in central and southern regions. In addition, our results showed a preferential north to south migratory dynamics and suggest a sex-biased dispersal in Mediterranean and southern regions. The estimated genetic patterns did not fit with the geographical relief of the Iberian landscape and they rather seem to follow political and linguistic territorial boundaries.

## Introduction

Recent approaches combining geographic information with genetic data provide robust analyses of the population structure across a landscape^1–6^. Essentially, these methods allow the study of patterns of spatially-explicit genetic differentiation by modulating genetic distances between samples or populations as a function of the geographic distance^1,2^.

Studies based on genome-wide autosomal data showed that genetic differentiation among European populations is, in general, highly correlated with geography^7,8^. However, genetic differentiation has also been associated with political-cultural boundaries that limit the gene flow between populations^8–10^. Previous studies have shown that major geographic features often lead to strong genetic differentiation at a global scale^11^, while at a regional/local scale cultural barriers usually modulate the genetic structure of populations^11–14^.

The Iberian Peninsula is separated from the rest of Europe by a range of mountains (the Pyrenees) and from Africa by a small stretch of water (the Strait of Gibraltar). The history of the Iberian Peninsula is characterized by multiple migrations and settlements from diverse population groups. The region was a refuge for populations fleeing from glaciers advance during the Last Glacial Maximum (LGM)^15,16^ and was probably a reservoir for the repopulation of Europe after the end of the LGM^17^. Recently, several studies based on ancient DNA shed new light into the genomic history of the Iberian Peninsula. Olalde *et al*.^18^ reported the presence of genetic structure in Palaeolithic hunter-gatherer populations. While the adoption of a sedentary lifestyle influenced the genetic variability of Iberians^19–21^, in the Neolithic individuals still carried a strong hunter-gatherer component due to admixture with hunter-gatherer populations^19,22,23^. Post Neolithic migrations also affected the genetic landscape of the region. A large replacement of male ancestry during the Bronze Age was inferred^24^ and it was recently associated with the massive immigration of steppe populations at ~4,500 years ago^25,26^. Additionally, two recent studies based on ancient DNA documented gene flow from North Africa toward the Iberian Peninsula at least since the Late Neolithic^18,27^. In the last two millennia, the Iberian Peninsula was occupied by Phoenicians, Greeks, Romans, German tribes and, more recently, most of the Peninsula was under Islamic rule (from the beginning of the 8^th^ until the end of the 15^th^ century) that was revoked with the so-called *Reconquista* where Catholic kingdoms recolonized the territory^28,29^. Current Iberian populations reflect this complex admixture of cultures and genetic backgrounds^9,30^, being one of the regions in Europe with the highest genetic diversity^31^. On the other hand, results based on autosomal markers showed a general homogeneity among Iberian populations^32^ with some local differentiation identified with mitochondrial DNA and Y-chromosome data^33–36^. Interestingly, previous studies showed that genetic variation correlates with the geographic distance in the Iberian Peninsula^37,38^. However, some of these studies were based on limited genetic information (for instance, only HLA genes). Altogether, the patterns of genetic variation across the Iberian Peninsula are still not totally clear and an analysis of comprehensive genetic data is required to address this issue.

Here we extended previous works by investigating genetic structure, genetic gradients and migratory dynamics of humans in the Iberian Peninsula at a fine-scale level through the analysis of a genome-wide dataset of 1,204 individuals belonging to 26 populations, based on current state of the art spatially-explicit models. Our results show that the genetic structure in northern Iberia agrees with the political frontiers established during the first centuries of the Reconquista, while the genetic landscape of central and southern regions do not show this association and present large migration corridors especially throughout the Mediterranean coast.

## Results

**Figure 1:**
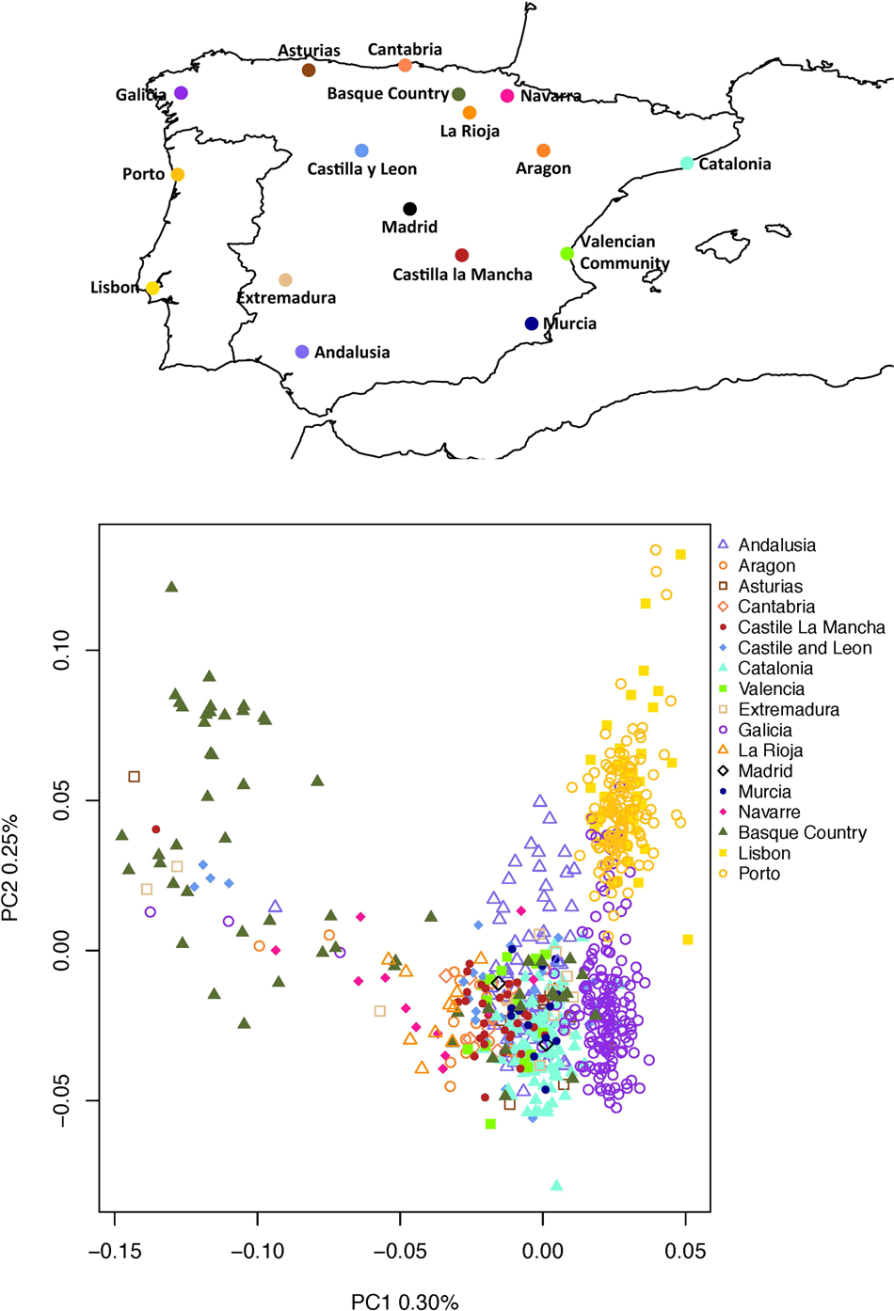
Geographic location of the 17 Iberian populations studied and population structure of the Iberian Peninsula. Geographic locations of the analysed data are shown above. The first and second principal components of the Iberian dataset are shown below.

We compiled genome-wide SNP data of individuals belonging to 26 populations from publicly available resources to generate two datasets: a global dataset with all the compiled populations (Table 1), and a second dataset (hereafter, Iberian dataset) that includes the 17 Iberian populations (Fig. 1). The global dataset was used for an exploratory analysis of the patterns of genetic variation and ancestral components in the Iberian Peninsula at the continental level, while the Iberian dataset was used for a fine-scale analysis of the genetic structure and heterogeneity in the Iberian Peninsula.

**Table 1:** Location, sample size and references of the datasets analysed in the present study.

Population	Sample size	Reference
Basque Country, Spain	57	^50, 51^
La Rioja	10	^51^
Navarre	12	^51^
Catalonia	93	^51^
Aragon	24	^51^
Valencia	16	^51^
Murcia	13	^51^
Andalusia	68	^30, 51^
Extremadura	14	^51^
Castile La Mancha	34	^51^
Madrid	2	^51^
Castile and Leon	36	^51^
Cantabria	10	^51^
Asturias	11	^51^
Galicia	177	^30, 51^
Porto	124	^49^
Lisbon	45	^49^
**Europe**		
Finland (FIN)	79	^53^
Northern and Western Europeans from Utah (CEU)	87	^53^
Tuscany, Italy (TSI)	96	^53^
Basque Country, France	24	^52^
**North Africa**		
Morocco South	16	^30^
Morocco North	18	^30^
Algeria	19	^30^
Tunisia	18	^30^
**Sub-Saharan Africa**		
Yoruba, Nigeria (YRI)	101	^53^

### High genetic similarity and mainly european ancestry in the Iberian Peninsula

In order to explore the presence of population stratification in global and Iberian datasets, we performed a principal component analysis (PCA). PCA results obtained from the global dataset showed a clear genetic differentiation between European, North African and sub-Saharan African populations for the first PC (PC1), while PC2 could distinguish between northern, central and southern Europeans (Supplementary Fig. S1a). In both PCs, Iberian populations cluster together. Interestingly, PC3 shows a cluster mainly composed by Iberian Basques and French Basques, both separated from the rest of the Iberian Peninsula (see Supplementary Fig. S1a). On the other hand, PCA results obtained from the Iberian dataset presented only a subtle genetic differentiation throughout the Iberian Peninsula (Fig. 1). In particular, PC1 separated most Basque samples from the rest of the populations and also, Porto, Lisbon, and some Andalusian samples appear separated from other Iberian populations. PC2 separated Portuguese, Galician and Andalusian samples from the rest of the Iberian samples (Fig. 1). Combining both PCs allows to obtain a global picture of genetic differentiation among the Iberian populations. PC3, PC4, and PC5 showed the inner diversity of Iberian populations while highlighting its global homogeneity (see Supplementary Fig. S1b).

We extended the analysis of population stratification by applying an unsupervised clustering algorithm (see Material and Methods) on the global dataset. Results showed that most ancestry of Iberians is shared with other European samples, followed by contributions from Africa (see Supplementary Fig. S2a). When applying the model with the lowest cross-validation error (K = 4) most Iberian individuals presented three main ancestral components (see Supplementary Fig. S2b). Two components are associated with European ancestry and one is associated with North African ancestry. It is noteworthy that for Basque individuals the North African ancestral component, which is present in the other Iberian samples, is only vestigial (see Supplementary Fig. S2a).

### Subtle genetic structure in the Iberian Peninsula based on the spatially explicit analysis

**Figure 2:**
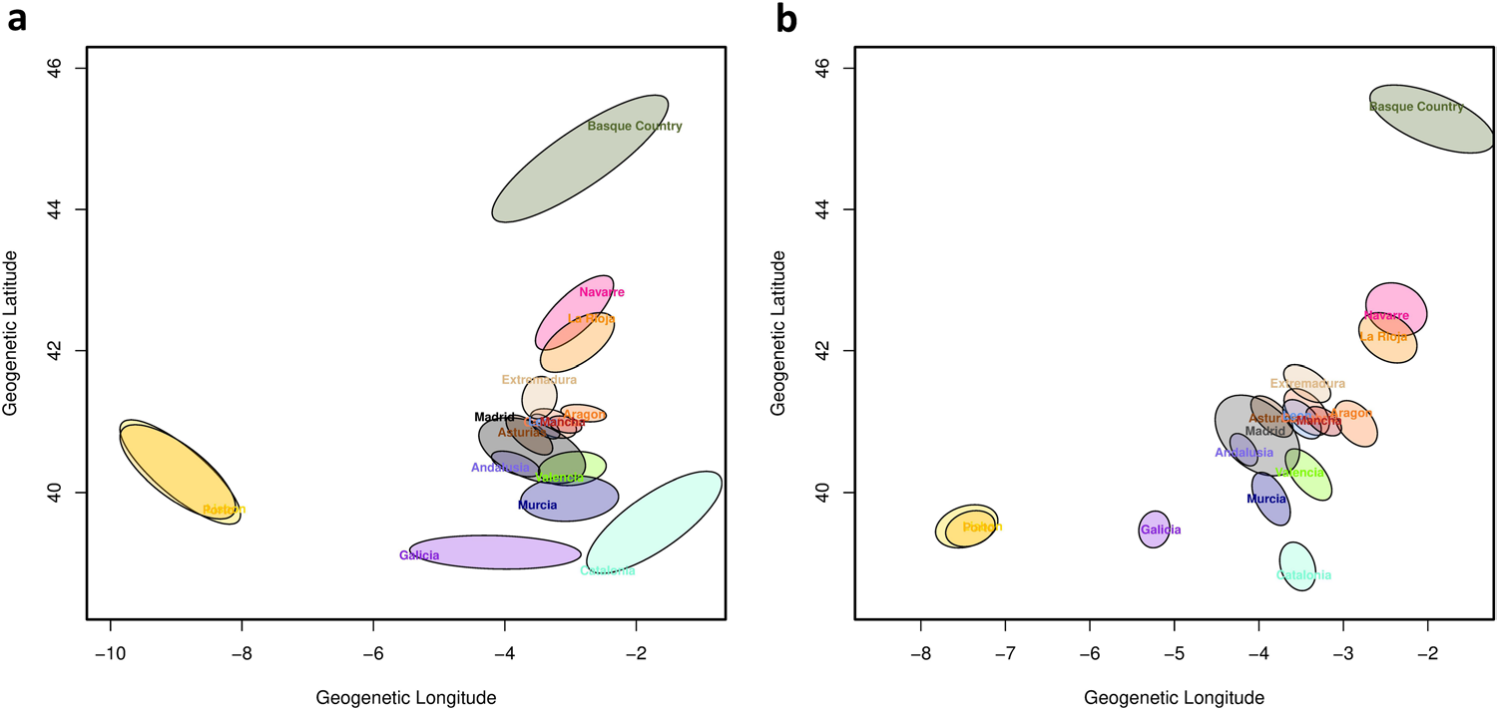
Geogenetic locations of the Iberian populations. The maps were inferred under (**a**) the model of isolation by distance with migration and (**b**) the model with migration and admixture estimated with SpaceMix. The plot shows the 95% confidence surfaces of the estimated geogenetic maps. Some names were abbreviated as Leon (Castile and Leon) and Mancha (Castile La Mancha).

We modelled the geographic structure of the Iberian Peninsula with the Bayesian framework included in the package SpaceMix (see Material and Methods). We found that isolation by distance models considering migration and migration with admixture presented a better fitting with the observed data, compared with models based on pure isolation by distance with and without admixture (see Supplementary Fig. S3). Geogenetic maps inferred under the best fitting models presented remarkable similarities (Fig. 2), which suggest that long-distance migrations followed by admixture events within the region were not a major contributor to the observed genetic structure in the Iberian Peninsula. Nevertheless, the 95% confidence interval ellipses inferred under the model of isolation by distance with migration and admixture (Fig. 2b) are smaller than the ellipses inferred under the model of isolation by distance with migration (Fig. 2a), meaning that allowing for long-range admixture more precisely delimited population location on the geogenetic map. Despite the improvement provided by considering long-distance admixture events, the estimated proportions of admixture in all populations were very low (<1%; see Supplementary Fig. S4). The geogenetic map inferred with the model of isolation by distance with migration and admixture presented 5 distinct population groups based on the 95% confidence surfaces (Fig. 2). The largest genetic divergence was observed between Portuguese and Basque Country populations. Indeed, our results highlighted some genetic isolation of the Galician population with respect to the other populations of the Iberian Peninsula, as well as a genetic isolation of populations of the Basque region (Basque Country, La Rioja and Navarre). The remaining Spanish populations (Northern, Central and Mediterranean) presented close geogenetic proximity, suggesting a high genetic similarity among them (Fig. 2).

**Figure 3:**
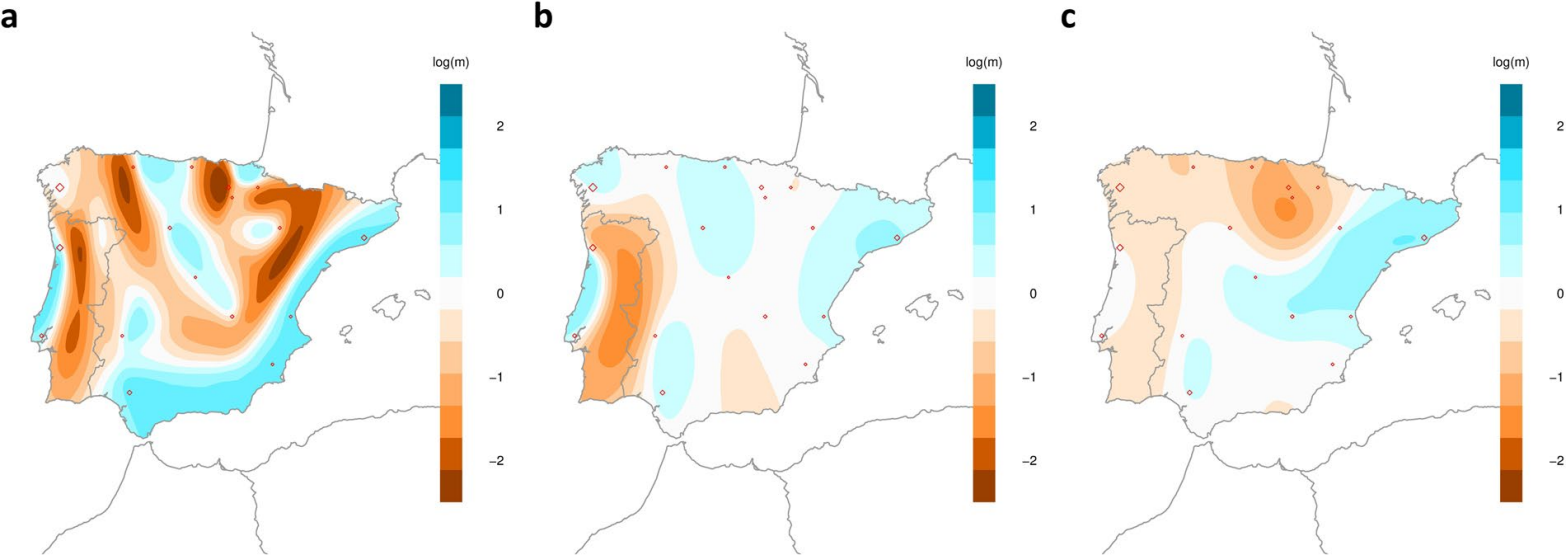
Effective Migration Maps estimated for the Iberian dataset. The maps were inferred from autosomes (**a**), X chromosome (**b**) and chromosome 7 (**c**). The plots were estimated with EEMS under a log_10_ scale and after mean centering. Blue regions indicate areas with effective migration rate higher than average (high genetic similarity), while brown colours indicate regions with a lower effective migration (compared to the average) between demes (high genetic differentiation).

The effective migration surface (EEMS) estimated from the autosomes presented several barriers to gene flow (regions of low effective migration rate) splitting the northern regions. It also showed corridors of genetic similarity (areas of high effective migration rate) connecting northern, central and southern regions (Fig. 3a). Populations from the Basque region appeared almost genetically isolated from the rest of the Peninsula. Also, Portuguese populations (southwest) were separated from central Iberian populations by a barrier to gene flow (see Supplementary Fig. S5). To a lesser extent, the region of Galicia presented some isolation from the rest of the Peninsula through barriers with Asturias and with the north of Portugal (see Supplementary Fig. S5). Another barrier was detected separating north and northeast regions (this is, separating Aragon and Catalonia) (see Supplementary Fig. S5). Concerning the opposite pattern (high genetic similarity), we inferred some regions with a high effective migration rate. One of them is a corridor throughout the Mediterranean coast, from the northeast to the south of the Iberian Peninsula. Another corridor presenting genetic similarity was identified connecting the central and northern coast of Portugal. A final migration corridor connected northern regions (Asturias and Cantabria) with central regions of the Iberian Peninsula (Castile and Leon, Madrid, Castile La Mancha) (Fig. 3a). The correlation between the estimated and observed genetic dissimilarities between and within demes (R^2^ coefficients of 0.80 and 0.95, respectively) suggested that the EEMS model was robust to describe the observed data (see Supplementary Fig. S6a, b). Indeed, the lack of correlation between geographic distance and genetic distance reveals that a model of isolation by distance cannot explain the population structure observed in the Iberian Peninsula (see Supplementary Fig. S6c).

Concerning the detection of putative sex-biased population structure in the Iberian Peninsula, we compared EEMS results from the autosomes with EEMS results from the X chromosome. EEMS results from the X chromosome revealed some different features concerning the genetic structure in this region, comparison with autosomes (Fig. 3b). Northern Iberian populations showed a stronger genetic structure (lower migration rates) than central and southern Spanish populations (higher migration rates). In agreement with the findings derived from autosomes, the analyses based on the X chromosome also indicated that the area with the highest genetic differentiation was the Basque region (see Supplementary Fig. S7). In particular, a strong barrier to gene flow surrounded the Basque Country and neighbouring populations. In contrast, a large region with high effective migration rate clusters populations from Eastern and Central regions of Iberia (Fig. 3b). Another region with high effective migration rate was identified, connecting the southern regions Andalusia and Extremadura (Fig. 3b). As for the autosomes, analyses based on the X chromosome showed that Portuguese populations are clustered together and are isolated from Spanish populations (Fig. 3b). Diagnostic plots for model fitting (see Supplementary Fig. S8a, b) showed that EEMS results present an excellent fitting with the data concerning dissimilarities within demes (R^2^ = 1.00) but the fitting was weak when considering dissimilarities between demes (R^2^ = 0.373). Similarly to the what was found with the autosomes, a significant deviation from an isolation by distance model (R^2^ coefficient = 0.01) was found when the observed pairwise genetic distances between populations were related with geographic distances (see Supplementary Fig. S8c). To test the robustness of the differences between X chromosome and autosomal analysis, we compared the differentiation patterns estimated for chromosome 7 with those from all the autosomes. Our results showed similarities in the patterns of genetic differentiation, even despite the poorer resolution due to the smaller amount of available data (Fig. 3c). Considering chromosome 7, Portuguese populations are genetically similar and isolated from the rest of Iberia (see Supplementary Fig. S9), and northern and central Iberian populations presented a high genetic similarity (Fig. 3c). Moreover, populations from the Basque region also showed higher degrees of genetic differentiation with respect to surrounding regions (Fig. 3c), in agreement with the analyses of all the autosomes (Fig. 3a). The most relevant difference between the results derived from chromosome 7 and from the autosomes is that chromosome 7 showed a barrier to gene flow separating Extremadura and Andalusia regions from other Mediterranean regions (Fig. 3c) which was not found in the analyses based on all the autosomes (Fig. 3 and see Supplementary Fig. S5). Diagnostic plots based on chromosome 7 showed that EEMS results present a reasonable fitting with the data in terms of dissimilarities within demes (R^2^ = 0.547) (see Supplementary Fig. S10a, b), but a weak fitting when exploring dissimilarities between demes (R^2^ = 0.107). Additionally, a deviation from an isolation by distance model (R^2^ = 0.074) was found when the observed pairwise genetic distances between populations were correlated with geographic distances (see Supplementary Fig. S10c). Taking into account the results from chromosome 7, we believe that our findings based on the X chromosome are not biased due to the smaller sequence length analysed but they should be interpreted with caution since the patterns found for the autosomes could only be partially replicated by the chromosome 7 analysis.

To further explore sex-biased patterns of genetic differentiation we applied the SpaceMix framework on the X chromosome data. However, none of the models implemented in this framework could accurately describe the pattern of decay of genetic covariance present in the observed data (results not shown).

## Discussion

The presence of genetic structure in the Iberian Peninsula has been described based on the Y chromosome and mtDNA at a regional level^9,33,36,39^. Here we extended those studies by considering a more comprehensive dataset of genome-wide genetic information, analysing autosomes and, for the first time, the X chromosome. We found that the characterization of the genetic landscape of the Iberian Peninsula, with spatially explicit approaches, presents subtle structure features. Patterns of genetic differentiation were largely observed along a longitudinal orientation (Fig. 3), in agreement with findings from other genetic markers^38^. Indeed, strong genetic differentiation was observed in northern regions of the Iberian Peninsula (Figs 2 and 3), while corridors of genetic similarity mainly appeared along a latitudinal orientation, along the Mediterranean and Atlantic shores (Fig. 3). Remarkably, we did not find an agreement between our results and the geographic relief of the Iberian landscape (see Supplementary Fig. S11), which suggests that geographic features did not have a major influence on the genetic patterns observed in Iberia. However, the patterns of genetic structure found in northern regions of the Iberian Peninsula are compatible with political and linguistic boundaries associated with the Catholic kingdoms formed during the first two centuries of the *Reconquista* (Fig. 3). This interpretation is in agreement with previous results based on haplotype data of Spanish samples^40^. However, the findings for northern regions contrasted with those for central and southern regions which presented a much more homogeneous genetic structure. In particular, we found that central and Mediterranean populations are genetically similar (Fig. 2) with only a barrier to gene flow separating Mediterranean and Extremadura populations from all the other Iberian populations (Fig. 3a). Interestingly, our results showed that Mediterranean populations present a high genetic similarity (see Supplementary Fig. S5), which is agreement with recent results by Olalde *et al*.^18^ showing that during the Roman period and onwards southern and Mediterranean populations had an influx of genes from southern Europe and North Africa, most likely reflecting the mobility by land and sea during the Roman empire^41^, the commercial trade across the Mediterranean Sea^29,42^ and the Islamic occupation of the Iberian Peninsula during 8 centuries^28^. In addition, our results for the X chromosome suggest that the current genetic structure in the Iberian Peninsula was influenced by sex-biased migrations, given that different genetic structures were found when analysing separately the X chromosome and the autosomes (e.g., absence of genetic differentiation between the regions of Catalonia and Aragon and lack of structure in Central and Southern regions of the Peninsula). However, as previously indicated, these results should be interpreted with caution since the patterns found for the autosomes could only be partially replicated when analysing only chromosome 7 markers, and further studies will be necessary to clarify this issue.

Populations from the Basque region (Basque Country, La Rioja and Navarre) showed a genetic distinctiveness from the other Iberian populations for both autosomes (Figs 1, 2a and S5) and X chromosome (Fig. 3b). This genetic differentiation could be caused by cultural aspects since Basques are characterized by their unique non-Indo-European language and limited gene flow from other populations outside Iberia such as north Africans, as shown in our analyses and also previously reported^43,44^. Additionally, the genetic similarity found between Spanish and French Basques using both PCA (Supplementary Fig. S1a) and ancestry profiles (Supplementary Fig. S2) could be explained by shared cultural traditions between those regions.

The northwest region of the Peninsula (Galicia) presented a higher than average genetic differentiation when compared to other Spanish populations (Figs 2 and 3). As for Basque populations, cultural and linguistic differences could account for this genetic divergence^45^. Moreover, a study on marital behaviour showed a high proportion of inbreeding that could lead to genetic differentiation from other Iberian populations^46^. Interestingly, Portugal and Galicia may share their ancestral history^40^ and we also found results supporting this hypothesis. The geogenetic map (Fig. 2) shows that Galicia is, genetically, the Spanish region closest to Portugal, despite the larger geogenetic distance estimated between Galicia and Portugal with respect to the distance between Galicia and the central regions of Spain. Additionally, the estimated effective migration surface for autosomes (Fig. 3) suggests a small Atlantic coastal corridor of gene flow connecting Galicia and northern Portugal, which can be attributed to the long historical relationship between these regions. Indeed, before the Islamic invasion in the 8^th^ century, both regions belonged to the Roman province of Gallaecia and later on to the kingdom of the Suebi (405 CE and 585 CE), before the annexation by the Visigoths^47,48^. Portugal became politically independent in 1143 and expanded rapidly toward the south (Portuguese *Reconquista* ended by 1249). The establishment of a political border could have promoted some cultural divergences but still important relationships were kept between Galicia and Portugal because of their geographic proximity, similar language and sociological factors. A recent study based on genome data but applying other approaches also showed remarkable genetic similarities between these regions^40^, in agreement with our findings.

In conclusion, we found that the genetic landscape across the Iberian Peninsula is complex, with contrasting patterns of remarkable genetic dissimilarity in the North and genetic homogeneity in the South. We interpret our findings considering that the geography is not the main factor shaping the genetic landscape of the Iberian Peninsula. Instead the major genetic dissimilarities estimated from our data better fitted with historical, political and cultural barriers that influenced migratory patterns and the relationships between populations..

## Materials and Methods

### Data and genotyping

We examined a genome-wide SNP dataset genotyped on the Affymetrix 6.0 chip, composed of 26 populations reported in several previous studies: 17 Iberian populations (2 Portuguese populations retrieved from Lopes *et al*.^49^ and 15 Spanish populations retrieved from Botigue *et al*.^30^, Henn *et al*.^50^ and Fernandez-Rozadilla *et al*.^51^), and 4 European populations (French Basques from the Human Genome Diversity Panel^52^, northern Europeans (CEU), Tuscans (TSI) and Finns (FIN) obtained from the 1000 Genomes Project^53^). Out of Europe we included 4 North African populations (Algeria, Morocco North, Morocco South and Tunisia) retrieved from Henn *et al*.^50^ and one sub-Saharan (Yoruba) from the 1000 Genomes Project^53^.

A quality control filter was applied using PLINK 1.9^54^. For each population, we excluded SNPs with missing genotype rate >10%, and those that failed Hardy-Weinberg equilibrium under a threshold of 0.05. We also excluded individuals with a missing rate >10% and those individuals that shared an identity-by-state >85%. In addition, after merging all populations, SNPs with a minor allele frequency (MAF) <0.05 were excluded. After applying quality control filters, the global dataset and the Iberian dataset presented a total of 1,204 and 746 individuals, respectively (Table 1). Additionally, SNPs were pruned using PLINK 1.9 with a sliding window of 50 kb, a shift step of 5 SNPs and a LD threshold of 0.5. We finally obtained a total of 64,302 and 174,001 SNPs for the global and the Iberian datasets, respectively (Supplementary Table S1). For the X chromosome, we excluded SNPs for both pseudoautosomal regions (PAR1 and PAR2) and SNPs heterozygous in the X specific region, keeping a total of 4,792 SNPs (Supplementary Table S1).

### Analysis of the population structure in the Iberian Peninsula

We performed the PCA with the *smartPCA* algorithm implemented in EIGENSOFT v5.0.1^55^ software. Additionally, we applied ADMIXTURE v1.3.0^56^ under unsupervised mode, testing from *K* = 2 to *K* = 10 ancestral clusters. We ran 10 replicates with different random seeds and merged the outputs from different runs. Results were then depicted with Distruct1.1^57^.

### Analysis of the spatial structure in the Iberian Peninsula

We investigated patterns of isolation by distance and gene flow throughout the Iberian Peninsula for the autosomes in the Iberian dataset with the Bayesian framework SpaceMix^1^. This analysis provides genetic relationships between populations through a geogenetic map in which geographical distances between populations are related with genetic distances. SpaceMix implements four different types of models: (1) a pure isolation by distance model, in which populations are stationary (absence of migration) and do not present admixture; (2) a model of isolation by distance with admixture, in which populations are stationary (absence of migration) and can present admixture; (3) a model of isolation by distance with migration, in which population locations are inferred (allowing migration) and cannot present admixture; (4) a model of isolation by distance with migration and admixture, in which population locations are inferred (allowing migration) and can present admixture. For each model we ran 10 independent short chains of 10^6^ iterations each, followed by a long chain of 10^7^ iterations based on the estimates of the last iteration of the short chain with the highest posterior probability. A sample was taken every 10^4^ iterations leading to a total of 1,000 points to estimate the posterior distribution of each parameter. Initial population locations were randomly taken from a uniform distribution of −180 to 180 and −90 to 90 for longitude and latitude, respectively.

We also identified patterns of spatial structure and genetic heterogeneity within the Iberian Peninsula applying the framework EEMS^2^ to the Iberian dataset. We estimated migration rate surfaces allowing the visualization of corridors and barriers to gene flow. Basically, EEMS considers the stepping-stone migration model to infer migration rates through a Bayesian approach^2^. The method applies a dense triangular grid that fills the entire landscape and assigns each individual to the geographic neighbour deme of each population to finally provide a map quantifying genetic dissimilarities. We also estimated a matrix of genetic dissimilarities between all 746 individuals with the *bed2cliffs* method implemented in the EEMS package. For all the analyses we specified a total of 1,000 demes and we performed 5 independent runs with 1.1 × 10^7^ iterations, a thinning interval of 1,000 iterations and 10^6^ iterations as burn-in. Finally, we adjusted migration and diversity parameters to model acceptance rates between 10–40% following the software documentation.

#### Sex-biased population structure

In order to test the presence of sex-biased migration in the Iberian Peninsula, we compared the patterns of genetic differentiation of the X chromosome with the autosomes applying the EEMS framework. Additionally, we analysed the patterns of genetic differentiation only on chromosome 7, which presents a size similar to the X chromosome (around 150 million bases), to ensure that the higher linkage on the X chromosome or the lower number of SNPs did not affect the results. Therefore we trimmed chromosome 7 to have a similar SNP density to the X chromosome (4,792 SNPs for the X chromosome and 4,755 SNPs for chromosome 7) by applying a LD threshold of 0.205 with PLINK 1.9 (Supplementary Table S1). We ran EEMS using the settings previously used to analyse the autosomes (see above).

## Supplementary information

Supplementary Material

## Data Availability

The data from North African populations and some Iberian populations can be found at t http://bhusers.upf.edu/dcomas/. The data from Portuguese and Spanish populations is available upon request to alopes@ipatimup.pt and angel.carracedo@usc.es, respectively.
