# Supplementary material for: Spatially explicit analysis reveals complex human genetic gradients in the Iberian Peninsula
Source: Sci Rep. 2019 May 24;9:7825. doi: 10.1038/s41598-019-44121-6 (PMC6534591; doi:10.1038/s41598-019-44121-6)
Supplement: Supplementary file 1 — Supplementary Material [file 41598_2019_44121_MOESM1_ESM.pdf]

Supplementary Material

Spatially explicit analysis reveals complex human genetic gradients in the Iberian Peninsula

João Pimenta, Alexandra M. Lopes, Angel Carracedo, Miguel Arenas, António Amorim, David Comas

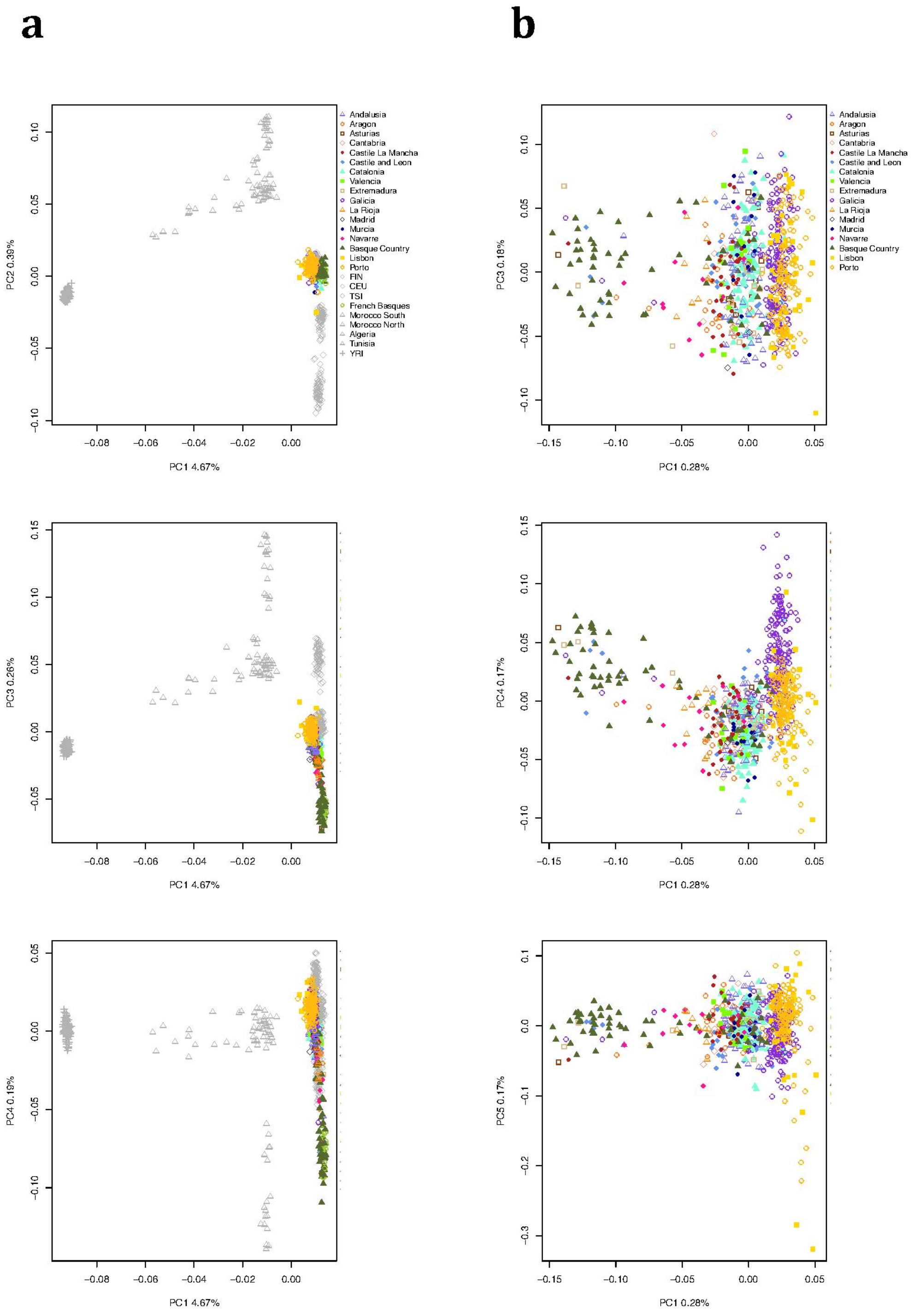

Figure S1. Population structure based on principal component analysis. (a) PC1 against PC2, PC3, PC4 for the dataset of European and Africans samples. (b) PC1 against PC3, PC4, PC5 for the Iberian dataset. Iberian samples and French Basques are colour-coded whereas the rest of samples are shown in grey.

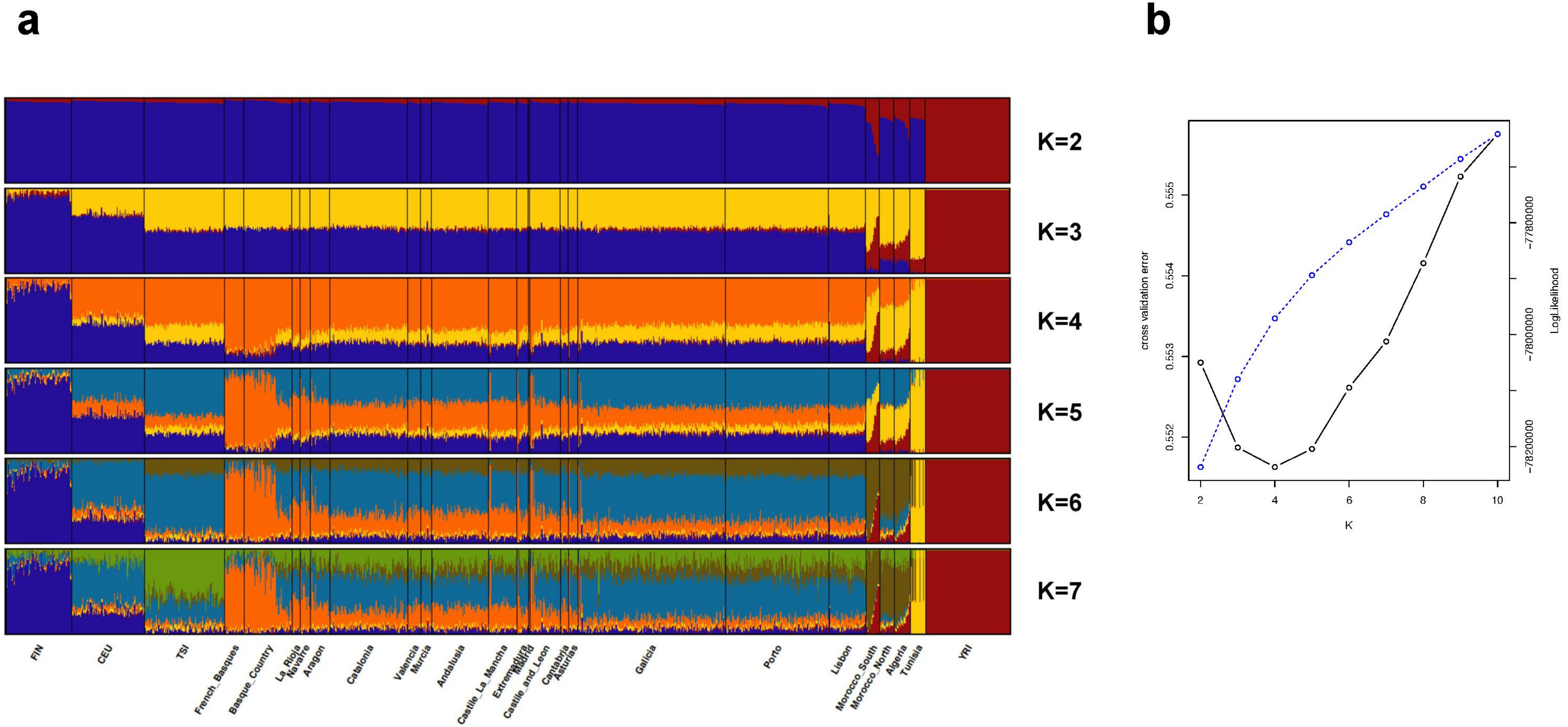

Figure S2. Ancestry inference estimated on the basis of model-based unsupervised clustering for the global dataset. (a) Ancestry plots for two to seven ancestral populations (K=2 to K=7) for all 26 populations included in the analysis. (b) Cross-validation (blue) and log-likelihood (black) for the ancestry analysis with K=2 to K=10. For K=4, the majority of Iberian ancestry is derived from a component largely present in

European populations (orange), while a Northern/Central European component (blue) is the second most relevant component in Iberian populations. A component associated with North Africa ancestry (yellow) was found as the third most relevant in Iberians and the sub-Saharan component (red) is only present at a vestigial level.

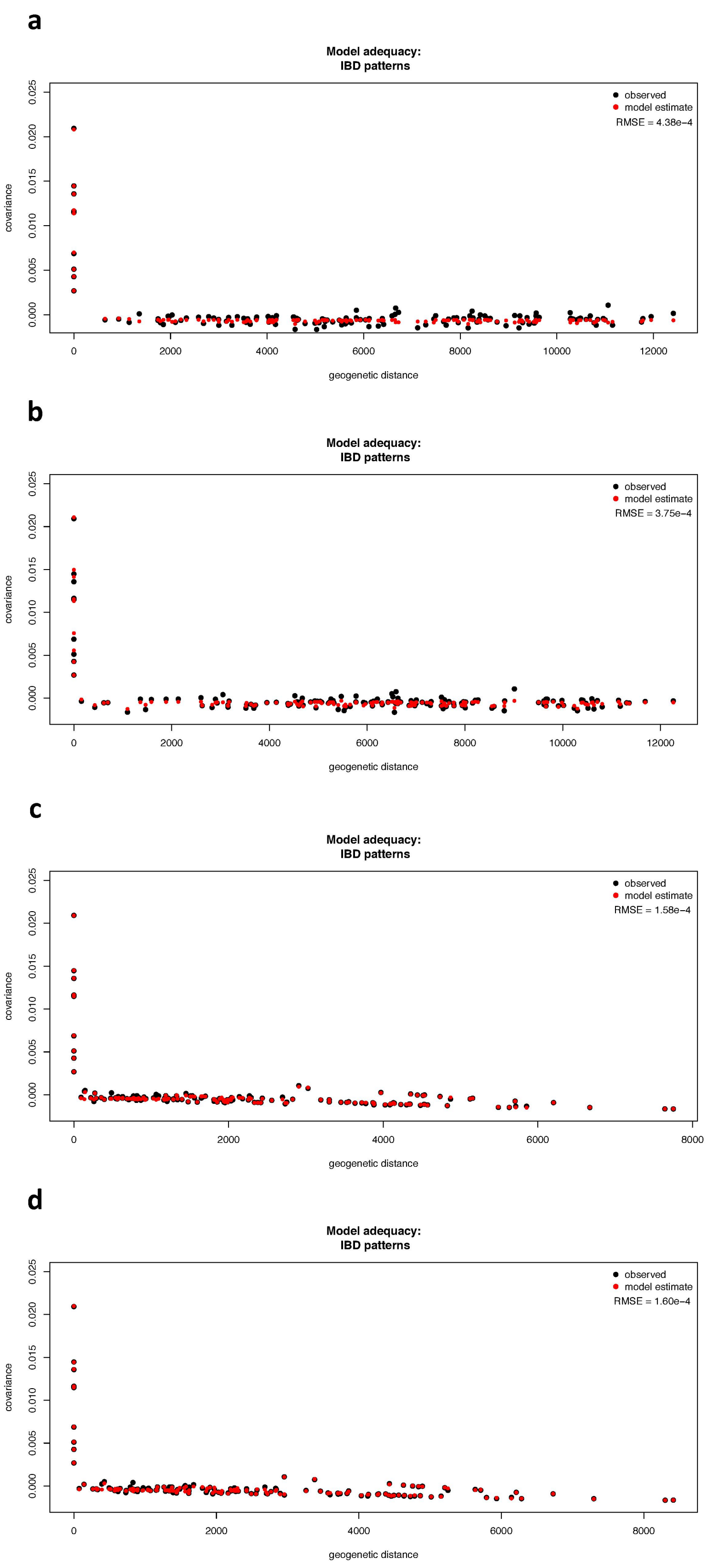

Figure S3. Fitting of the models with the observed data through the SpaceMix framework. Each plot shows the patterns of decay of covariance estimated for the observed geographic (black) and the inferred geogenetic (red) distances. The plots show the following analyzed models: a) No Movement: populations do not choose

their location and are not able to draw admixture, b) Source: populations do not choose their locations but are able to draw admixture, c) Target: populations choose their location but are not able to draw admixture, d) Source and Target: populations choose their location and are able to draw admixture. For each model we estimated the Root Mean Square Error (RMSE) (see legend of plots) and we found that models “c” and “d” better fit with the real observations.

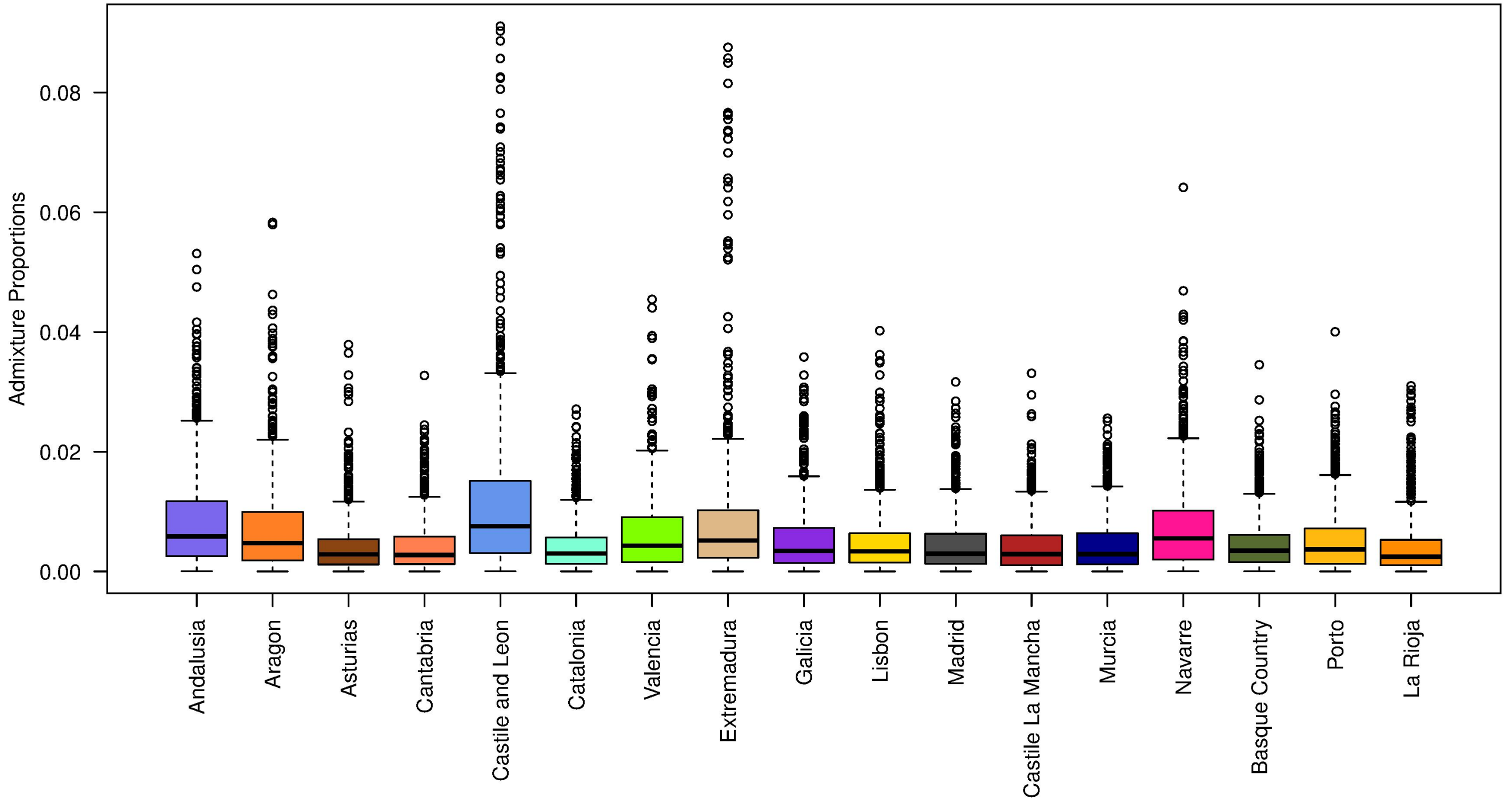

Figure S4. Admixture proportions inferred by SpaceMix for the isolation by distance with migration and admixture model. Boxplot of the admixture proportions inferred with SpaceMix for each Iberian population**.**

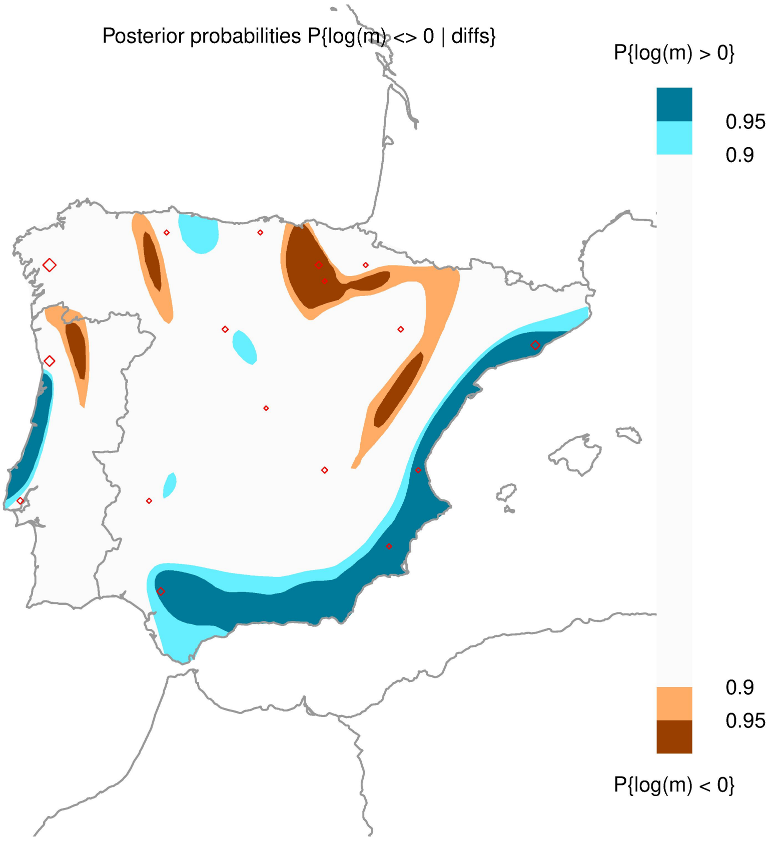

Figure S5. Corridors and barriers to gene flow estimated for autosomes of the Iberian dataset. The map highlights regions with a migration rate significantly high (blue) or low (brown) relative to the estimated mean.

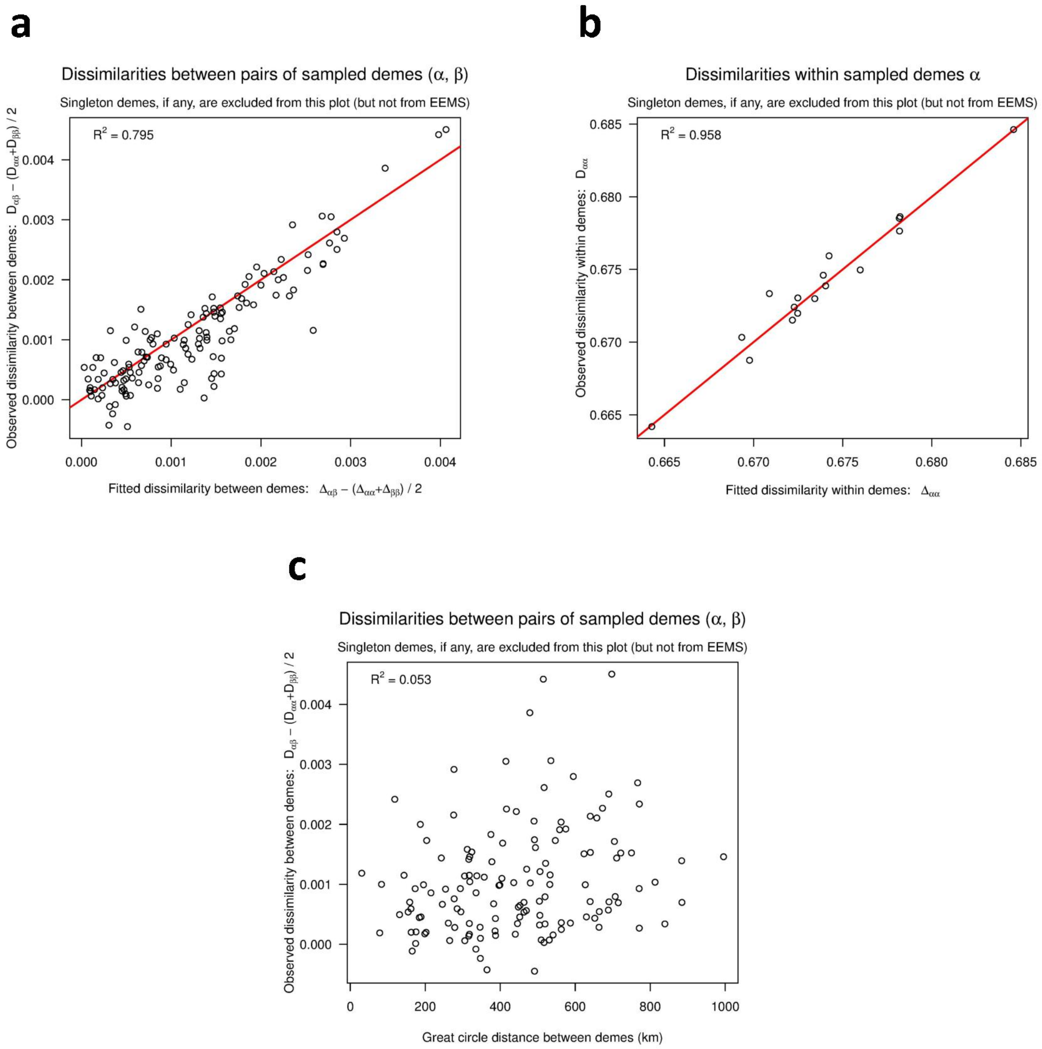

Figure S6. Diagnostic plots for EEMS model fitting for the autosomes. (a) Pairwise comparison of estimated and observed genetic dissimilarities between demes. (b) Pairwise comparison of estimated and observed genetic dissimilarities within demes. Note that the EEMS model presents an acceptable fitting with the observed data. (c) Scatter plot of observed genetic distances with geographic distances between populations. The R^2 coefficient (shown at the top left of each plot) was estimated for each scatterplot.

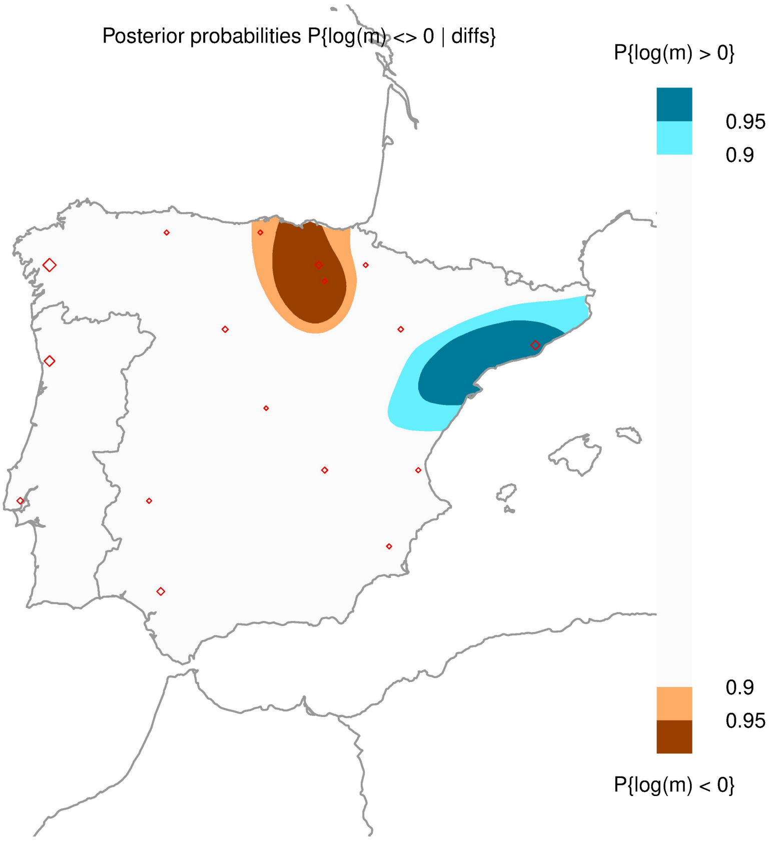

Figure S7. Corridors and barriers to gene flow based on the X chromosome. The map highlights regions with a migration rate significantly high (blue) or low (brown) relative to the estimated mean.

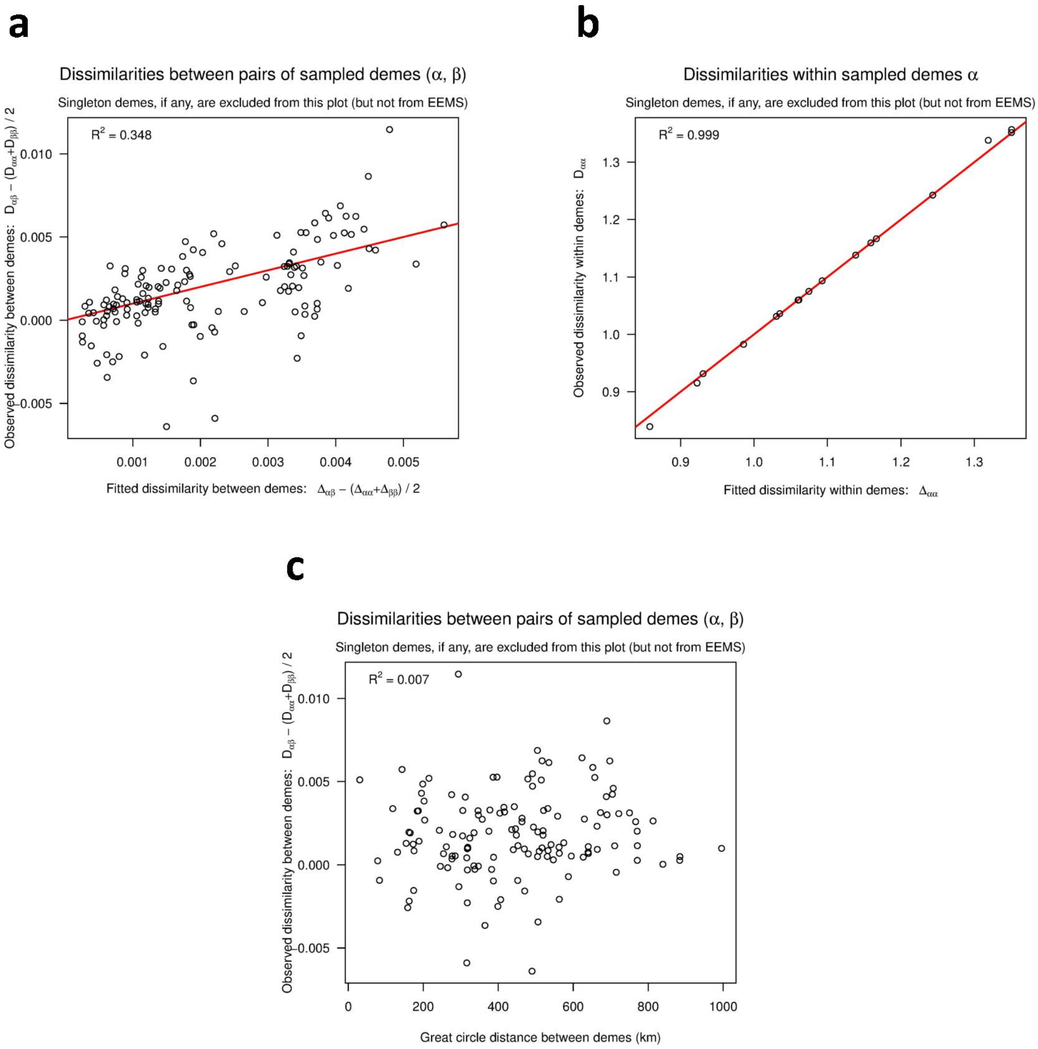

Figure S8. Diagnostic plots for EEMS model fitting for the X chromosome. (a) Pairwise comparison of estimated and observed genetic dissimilarities between demes. (b) Pairwise comparison of estimated and observed genetic dissimilarities within demes. Note that the EEMS model presents an acceptable fitting with the observed data. (c) Scatter plot of observed genetic distances with geographic distances between populations. The R^2 coefficient (shown at the top left of each plot) was estimated for each scatterplot.

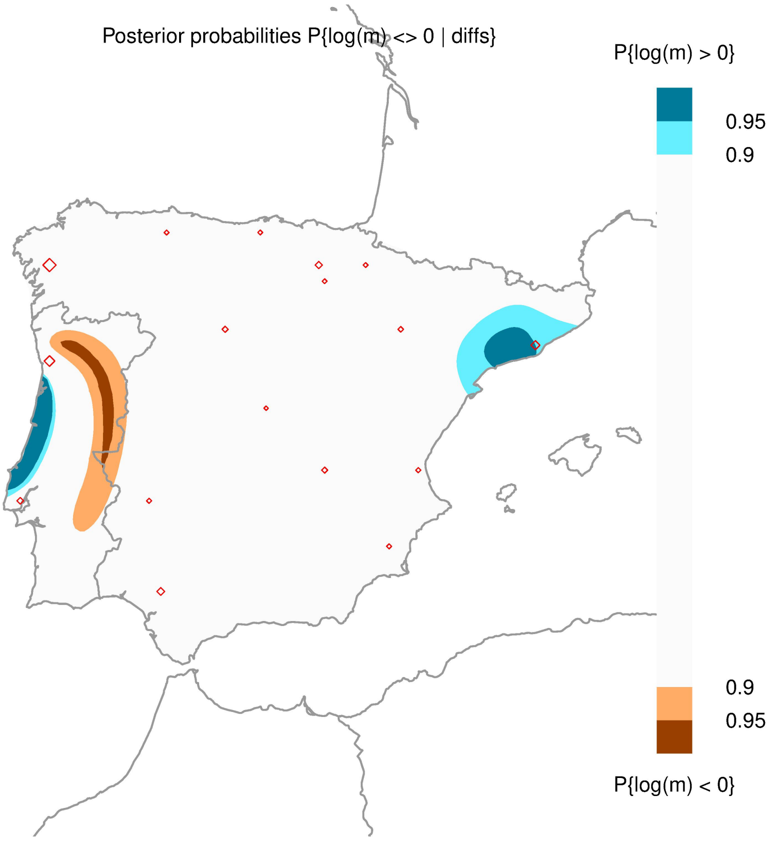

Figure S9. Corridors and barriers to gene flow based on chromosome 7. The map highlights regions with a migration rate significantly high (blue) or low (brown) relative to the estimated mean.

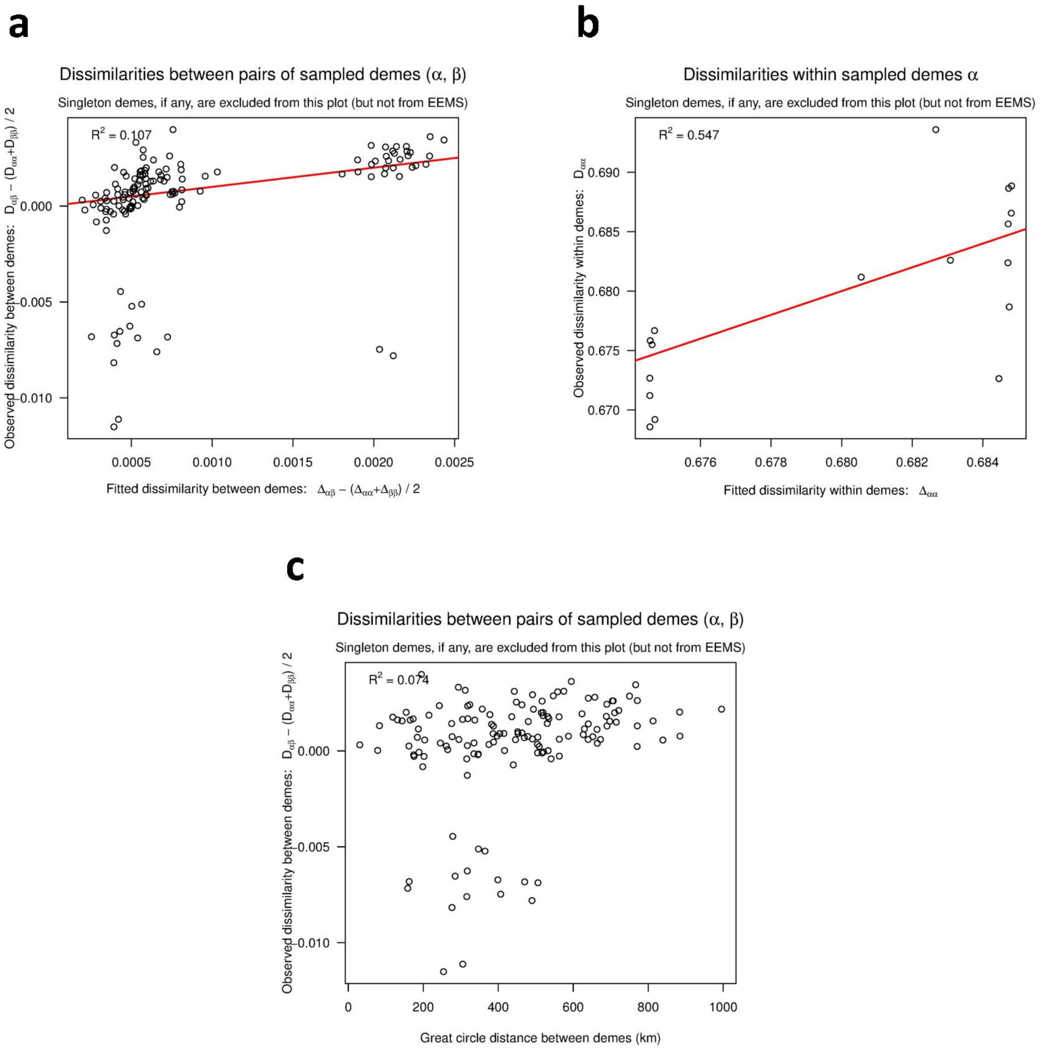

Figure S10. Diagnostic plots for EEMS model fitting for chromosome 7. (a) Pairwise comparison of estimated and observed genetic dissimilarities between demes. (b) Pairwise comparison of estimated and observed genetic dissimilarities within demes. Note that the EEMS model presents an acceptable fitting with the observed data. (c) Scatter plot of observed genetic distances with geographic distances between populations. The R^2 coefficient (shown at the top left of each plot) was estimated for each scatterplot.

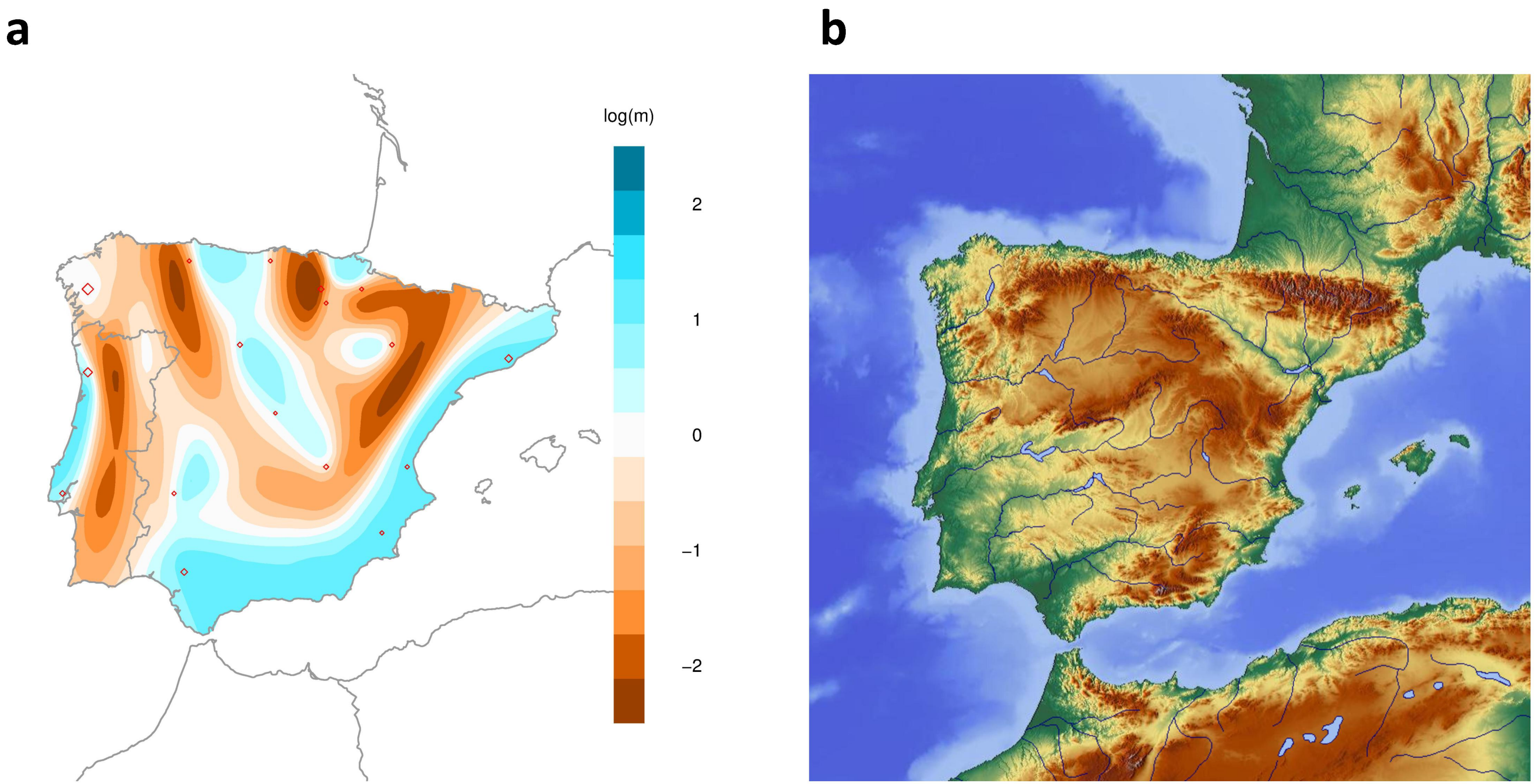

Figure S11. Comparison between the effective migration surface inferred for the autosomes and the geographical relief of the Iberian Peninsula landscape. The relief map was retrieved from OpenStreetMap website (© OpenStreetMap contributors).

Table S1. Number of SNPs and individuals for the studied datasets.

Global Dataset - autosomes		
**Filter**	**Number of SNPs**	**Number of Individuals**
**Merge dataset - LD pruning (0.5)**	64,302	1,204

Iberian Dataset - autosomes		
**Filter**	**Number of SNPs**	**Number of Individuals**
**Merge dataset - LD pruning (0.5)**	174,001	746

Iberian Dataset - X chromosome		
**Filter**	**Number of SNPs**	**Number of Individuals**
**Merge dataset - LD pruning (0.5)**	4,792	746

Iberian Dataset - chromosome 7		
**Filter**	**Number of SNPs**	**Number of Individuals**
**Merge dataset - LD pruning (0.205)**	4,755	746
